# Supplementary material for: Distinct medical and substance use histories associate with cognitive decline in Alzheimer’s Disease
Source: medRxiv. 2024 Nov 28:2024.11.26.24317918. Preprint. [Version 1] doi: 10.1101/2024.11.26.24317918 (PMC11623748; doi:10.1101/2024.11.26.24317918)
Supplement: Supplement 1 [file media-1.pdf]

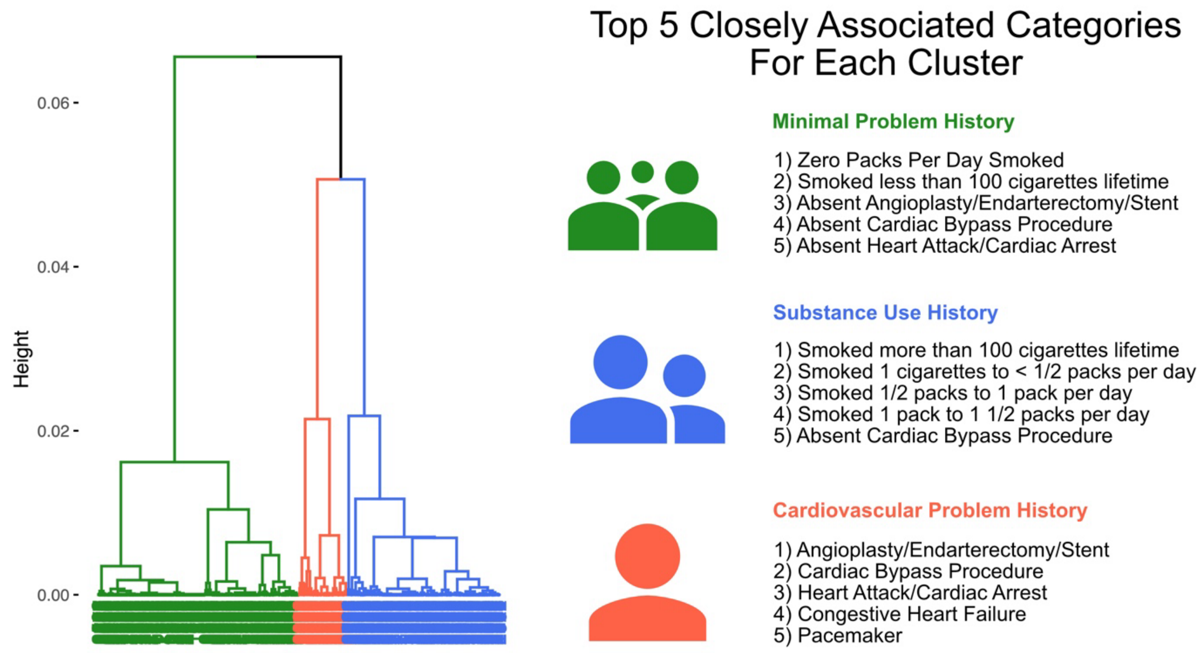

Supplementary Figure 1: Dendrogram from Clustering Analysis. The height of each branch represents the relationship between individuals in the dataset. The top five most closely associated problem history items are shown and were used to inform the naming of each cluster.
