# Supplementary material for: Distinct medical and substance use histories associate with cognitive decline in Alzheimer’s Disease
Source: medRxiv. 2024 Nov 28:2024.11.26.24317918. Preprint. [Version 1] doi: 10.1101/2024.11.26.24317918 (PMC11623748; doi:10.1101/2024.11.26.24317918)
Supplement: Supplement 2 [file media-2.pdf]

Supplementary Table 1. Pairwise Comparisons of Problem History Clusters on Clinical Dementia Rating Sum of Boxes at NACC Visit 3

	Contrast (95% CI)	p value
Minimal – Substance Use	0.11 (-0.01 - 0.23)	0.067
Minimal – Cardiovascular History	-0.15 (-0.32 - 0.03)	0.098
Substance Use – Cardiovascular History	-0.25 (-0.43 - -0.08)	0.005

Pairwise comparisons between each problem history cluster on the Clinical Dementia Rating Sum of Boxes (CDRSUM) at visit 3 adjusting for age, sex, *APOE* genotype, baseline CDRSUM, and visit number. The reported estimates reflect the adjusted mean difference in CDRSUM between clusters with 95% confidence intervals and p values representing statistical significance.
